# Population-Based Evidence of Familial Clustering in Pulmonary Carcinoid Tumors: Insights From the Utah Population Database

**DOI:** 10.7759/cureus.94208

**Published:** 2025-10-09

**Authors:** Sikandar Ansari, Muhammad Abbas Raza, Victor Perez-Gutierrez, Areeba Ahmer, Lisa Cannon-Albrigth

**Affiliations:** 1 Interventional Pulmonology, Huntsman Cancer Institute, University of Utah, Salt Lake City, USA; 2 Medical School, Aga Khan University, Karachi, PAK; 3 Genetic Epidemiology and Internal Medicine, Huntsman Cancer Institute, University of Utah, Salt Lake City, USA

**Keywords:** familial clustering, genealogy, neuroendocrine tumors, pulmonary carcinoid, utah population database

## Abstract

Background: Pulmonary carcinoid tumors are rare neuroendocrine neoplasms. While gastrointestinal carcinoids have established familial clustering, the heritable component of pulmonary carcinoids, particularly outside of multiple endocrine neoplasia type 1 (MEN1) syndrome, remains poorly defined. Utah's unique linked genealogy-cancer registry enables population-based assessment of familial risk.

Methods: We used the Utah Population Database (UPDB) linked to the Utah Cancer Registry (a Surveillance, Epidemiology, and End Results (SEER) registry) to identify individuals diagnosed with pulmonary carcinoid tumors (International Classification of Diseases for Oncology, Third Edition (ICD-O-3), histology codes 8240, 8249). The Genealogical Index of Familiality (GIF) test evaluated excess pairwise relatedness compared with 1,000 matched control sets. Relative risks (RRs) were estimated for first-, second-, and third-degree relatives, with expected cases based on cohort-specific incidence rates.

Results: A total of 232 pulmonary carcinoid cases with ≥3 generations of genealogy were identified. The GIF test demonstrated significant excess relatedness (GIF: 5.36 vs. 2.65; p=0.002), with excess in parent-offspring and avuncular pairs. RRs were significantly elevated for first-degree relatives (RR: 5.78; 95% CI: 1.03-18.21; p=0.048) and second-degree relatives (RR: 12.91; 95% CI: 6.42-23.29; p<0.0001), but not third-degree relatives. We identified 61 high-risk pedigrees with significantly more cases than expected. Incidence rates of pulmonary carcinoid were similar in Utah (0.8%) and the rest of SEER (0.7%), despite markedly lower overall lung cancer incidence in Utah.

Conclusions: Our findings demonstrate the significant familial clustering of pulmonary carcinoid tumors in a large, population-based resource, supporting a potential heritable contribution independent of MEN1 syndrome. These results justify further genetic and environmental investigations in high-risk pedigrees to identify susceptibility loci and modifiable exposures.

## Introduction

Over the past decade, substantial advances in the understanding and treatment of lung cancer, particularly non-small cell lung cancer (NSCLC), have led to the development of targeted therapies and immunotherapies that have significantly improved patient outcomes [1].

Neuroendocrine tumors classified as carcinoids occur infrequently, with the gastrointestinal tract being the most common site of origin, followed by the lungs; involvement of other organs is comparatively uncommon. The well-known carcinoid syndrome, characterized by episodic flushing, hypotension, bronchospasm, abdominal cramps, and diarrhea, occurs in a minority of gastrointestinal carcinoid cases, largely due to the secretion of vasoactive substances such as serotonin, bradykinin, and prostaglandins. In primary pulmonary carcinoid tumors, carcinoid syndrome is rare (occurring in less than 3% of cases), as these tumors typically produce lower levels of vasoactive agents, many of which are inactivated in the lung [2].

Histologically, pulmonary carcinoids are divided into typical and atypical forms. Typical carcinoids generally display low-grade features, slow growth, and limited metastatic risk, while atypical carcinoids are intermediate-grade tumors with higher invasive and metastatic potential; both comprise a spectrum of lung neuroendocrine tumors that includes large cell neuroendocrine carcinoma and small cell lung cancer at the high-grade end [3]. The etiology of pulmonary carcinoid tumors remains poorly understood. Unlike other lung cancers, typical carcinoids are not associated with smoking, while atypical carcinoids may have a weak, inconsistent association. Germline predisposition to carcinoid development is well-recognized in multiple endocrine neoplasia type 1 (MEN1) syndrome, although pulmonary involvement is rare compared to gastrointestinal or thymic carcinoids; prevalence estimates vary and remain low, one to two cases per 100,000 individuals per year [4].

Evidence for familial clustering of carcinoid tumors is stronger for gastrointestinal sites: reports include aggregation among first-degree relatives even in the absence of MEN syndromes. Familial pulmonary carcinoid tumors have only been described in rare case reports and limited registry-based studies. The Utah Population Database (UPDB), a unique multigenerational genealogical resource linked to a high-quality, statewide Surveillance, Epidemiology, and End Results (SEER) cancer registry, provides an unprecedented opportunity to investigate familial risk for this rare malignancy in a defined population [5]. Given that neuroendocrine differentiation and shared genetic pathways (such as dysregulation of cell-cycle control and chromatin remodeling genes) are implicated across gastrointestinal and pulmonary carcinoids, it is biologically plausible that mechanisms driving familial clustering in gastrointestinal sites could also extend to the lung. This study aimed to describe the incidence of pulmonary carcinoid tumors in Utah and to assess familial clustering using the UPDB, testing the hypothesis that a subset of pulmonary carcinoid tumors has a heritable component independent of MEN1 syndrome. If such a heritable risk is confirmed, these findings could inform genetic counseling, guide targeted surveillance strategies in high-risk families, and ultimately contribute to earlier detection and improved outcomes for this rare malignancy.

## Materials and methods

UPDB 

The UPDB resource allows consideration of the familial clustering of individuals diagnosed with pulmonary carcinoid tumors in Utah. The resource consists of the computerized genealogy of a majority of the Utah population from its founders in the mid-1800s to the present day. Approximately 2.7 million individuals are part of three or more generations of data; over 148,800 of these individuals have linked cancer diagnosis data from the Utah Cancer Registry (UCR). The UCR, founded in 1966, has tracked all cancers diagnosed or treated within the state. By 1973, it was incorporated into the SEER program of the National Cancer Institute (NCI) as one of its earliest registries. All independent primary cancers are recorded, with data including primary site, histology, behavior, and survival data, among others. The UCR patient data are linked to the UPDB genealogy, allowing the consideration of all genetic relationships among cancer cases.

Identification of pulmonary carcinoid cancer cases 

Pulmonary carcinoid cancer cases were identified by primary site, lung or bronchus, with histology codes 8240 or 8249, according to the International Classification of Diseases for Oncology, Third Edition.

Analysis of familial clustering 

To determine whether pulmonary carcinoid cases occurred in families more often than expected by chance, we used the Genealogical Index of Familiality (GIF), a method that quantifies excess relatedness within the UPDB. The GIF has been applied for decades to describe familial clustering in Utah [6,7]. This test calculates the average pairwise relatedness among all possible case pairs using the Malécot coefficient of kinship, which estimates the probability that two randomly sampled genes, one from each individual, are identical by descent from a shared ancestor.

**Figure 1:**
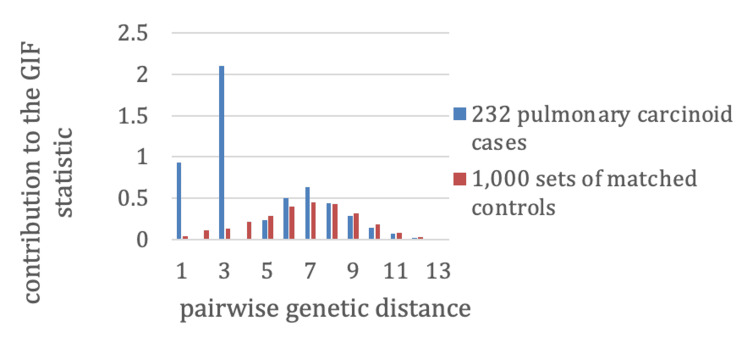
Familial clustering of pulmonary carcinoid tumors by genetic distance Contributions to the GIF are shown for 232 pulmonary carcinoid cases (blue) and 1,000 matched control sets (red). Stronger clustering is observed among close relatives, particularly parent-offspring (distance=1) and avuncular (distance=3) pairs, suggesting possible hereditary or shared environmental influences. GIF: Genealogical Index of Familiality

The analysis compares the observed average relatedness among pulmonary carcinoid cases with the expected relatedness from control groups randomly drawn from the UPDB. Controls were matched to cases by sex, birth year (within five years), and birth state, with 1,000 matched sets generated to estimate expected relatedness (Figure 1). Contributions to the overall GIF statistic were also stratified by genetic distance: one for parent-offspring, two for siblings or grandparent-grandchild, three for avuncular pairs (aunt/uncle-niece/nephew), four for first cousins (or equivalent), and six for second cousins (or equivalent).

Relative risks (RR) in relatives 

RR is the standard measure used to assess genetic contributions to disease. It compares the observed number of affected relatives, by relationship type, with the number expected based on population disease rates. To generate population-based rates for pulmonary carcinoid cancer, all individuals in the UPDB were grouped into cohorts defined by sex, five-year birth year intervals, and birth state (Utah vs. non-Utah). For each cohort, the rate of pulmonary carcinoid cancer was calculated as the number of cases divided by the total number of individuals.

The observed count of affected relatives for each relationship group was determined without duplication. Expected counts were then calculated by applying the cohort-specific rates to all relatives of the cases. RR was obtained by dividing observed by expected case counts. Assuming the observed distribution of pulmonary carcinoid cases follows a Poisson distribution with a mean equal to the expected count, 95% confidence intervals for RR were computed following the method of Agresti and Min [8].

High-risk pulmonary carcinoid pedigrees 

Using the UPDB, we identified all clusters of pulmonary carcinoid cancer cases that trace back to a common ancestor. These clusters, or pedigrees, may overlap, since individuals can belong to more than one cluster. To evaluate whether a pedigree represented a high-risk family, we compared the observed number of affected descendants with the number expected. Expected case counts were calculated by summing the cohort-specific incidence rates of pulmonary carcinoid cancer across all descendants (as described above). A pedigree was classified as high risk if the observed number of cases significantly exceeded expectation, defined as p<0.05. Significance testing was performed independently for each pedigree using a Poisson-based model without formal adjustment for multiple comparisons, consistent with prior UPDB analyses. This approach prioritizes sensitivity in detecting potentially informative high-risk families, which can then be evaluated in subsequent genetic studies.

## Results

Incidence of pulmonary carcinoid

In Utah, the incidence of pulmonary carcinoid was 0.8% (95% CI: 0.7-0.9), and of other lung cancers, it was 27.9% (95% CI: 27.3-28.4). In all the other states with SEER-reported data, excluding Utah, the incidence of pulmonary carcinoid was 0.7% (95% CI: 0.7-0.8), and the incidence of other lung cancers was 60.7% (95% CI: 60.6-60.8).

Analysis of familial clustering 

Using the Utah genealogy data linked to the Utah NCI SEER cancer registry, 232 pulmonary carcinoids were diagnosed in individuals with at least three generations of genealogy. Table 1 shows the GIF test for excess relatedness, including the number of cases (n), case GIF statistic, mean control GIF statistic, significance for the GIF test, case distant GIF (dGIF) statistic, mean control dGIF statistic, and significance for the dGIF test. Figure 1 shows the contribution to the GIF statistic by pairwise genetic distance for cases compared to the mean results for 1,000 sets of matched controls. As seen in Figure 1, there is a clear excess of parent-offspring pairs of cases (pairwise genetic distance=1), as well as a clear excess of pairwise genetic distance=3, which represent avuncular pairs. No pairs of affected siblings or grandparent-grandchild pairs were observed (pairwise genetic distance=2); neither were any affected first cousins observed (pairwise genetic distance=4). It is a little unusual to observe more pairs where members are in different generations (e.g., parent-offspring, distance=1) than pairs whose members are in the same generation (e.g., siblings, distance=2); however, the sample size of pulmonary carcinoid cases is very small (n=232), and few pairs of any relationship are expected.

**Table 1:** GIF analysis of familial clustering for 232 cases of pulmonary carcinoid cancer GIF measures overall average relatedness between all pairs of cases; higher values indicate greater familial clustering than expected by chance. dGIF excludes close relationships to assess more distant familial aggregation. Significance was assessed by comparison to 1,000 matched control sets. *P-value is considered significant if p<0.05. GIF: Genealogical Index of Familiality; dGIF: distant GIF

Probands	n	Case GIF	Mean control GIF	P-value	Case dGIF	Mean control dGIF	P-value
Pulmonary carcinoid cases	232	5.36	2.65	0.002*	2.33	2.38	0.508

RR in relatives 

Table 2 shows the estimated RRs for pulmonary carcinoid cancer cases among the first-, second-, and third-degree relatives of the 232 cases including the relative type, the number of relatives (n), the number of pulmonary carcinoid cancers observed among relatives (0bs), the number of pulmonary carcinoid cancers expected among relatives (exp), the estimate RR, the one-tailed significance (p-value), and the 95% confidence interval for the RR (95% CI). 

**Table 2:** Estimated RR for pulmonary carcinoid cancers among the relatives of the 232 individuals diagnosed with pulmonary carcinoid cancer RRs were calculated by dividing the observed number of affected relatives by the expected number, based on cohort-specific incidence rates. Confidence intervals assume a Poisson distribution. *Significance was defined as p<0.05. RRs: relative risks

Relationship	n relatives	Observed cases (Obs)	Expected cases (Exp)	RR	95% CI	P-value
First degree	2,059	2	0.4	5.78	1.03-18.21	0.048*
Second degree	6,802	8	0.6	12.91	6.42-23.29	<0.0001*
Third degree	17,682	1	1.6	0.62	0.03-2.93	0.519

High-risk pulmonary carcinoid pedigrees 

**Figure 2:**
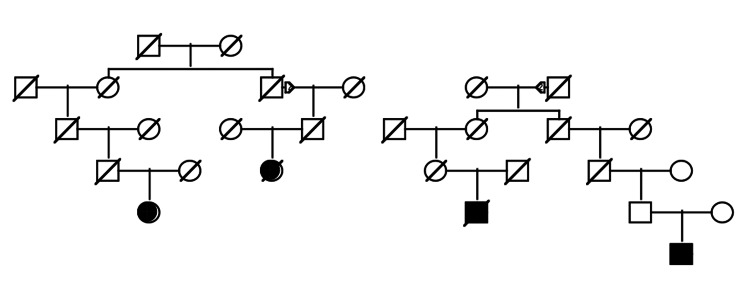
An example of a Utah high-risk pulmonary carcinoid cancer pedigree An example of a high-risk pedigree with four pulmonary carcinoid cases among descendants of a founder born in the 1830s. The observed-to-expected case ratio was 4/0.6 (p=0.003). Squares (□): male individuals; circles (○): female individuals; diagonal slash (∕): deceased individual; filled symbol (● or ■): affected individual (in this case, diagnosed with pulmonary carcinoid tumor); unfilled symbol: unaffected individual (no known pulmonary carcinoid); triangles (▵): represent founders or ancestral couples in earlier generations

The 232 pulmonary carcinoid cancer cases clustered into 110 non-overlapping pedigrees, including two to six related pulmonary carcinoid cases. Each pedigree was tested for a significant excess of cases among the descendants using rates estimated as described above; 61 pedigrees, including two to four related cases, were observed to have significantly more cases among descendants than expected using UPDB cohort-matched rates. Figure 2 shows an example of a high-risk pulmonary carcinoid cancer pedigree with four cases among the descendants. The founder of the UPDB was born in the eastern United States in the early 1830s and had more than 11,600 descendants in UPDB (not shown); a total of four pulmonary carcinoid cancers (shown as fully shaded) were observed among the descendants, with 0.6 expected (p=0.003). The son in the second generation has offspring with two different partners.

## Discussion

After gastrointestinal carcinoid tumors, pulmonary carcinoid tumors are the most common type of carcinoid tumors [9]. While rare, their incidence has been increasing over the last 40 years, thought in part to be due to the increased diagnosis of early-stage tumors because of advances in radiographic and endoscopic techniques [10]. In clinical and histological terms, pulmonary carcinoids are categorized as typical or atypical. The typical subtype tends to progress slowly and rarely metastasizes, whereas atypical tumors often behave more aggressively [5]. Tumors localized to the airways usually present with signs and symptoms of airway obstruction, such as cough, dyspnea, wheezing, hemoptysis, and recurrent pneumonia, whereas many peripheral parenchymal tumors are asymptomatic and only discovered incidentally on imaging studies. Rarely, pulmonary carcinoid tumors can also present as paraneoplastic syndromes. The classic "carcinoid syndrome", however, is present in less than 1% of patients at presentation. Cushing syndrome is more common, with bronchopulmonary carcinoid tumors being the most common site of ectopic adrenocorticotropic hormone (ACTH) production, but still relatively rare overall, occurring in about 4% of patients [11]. 

The exact etiology of carcinoid tumors is unknown. Pulmonary carcinoids do not appear to be associated with the same environmental risk factors, such as smoking and radiation exposure, that are typically thought of as being causative of other types of lung cancer [12]. There is a known genetic link to the development of pulmonary carcinoids (as well as thymic and small intestinal carcinoids) in association with the MEN1 syndrome, though the majority of these tumors are felt to be sporadic in nature [5]. Even among sporadic carcinoids in patients without other features suggestive of MEN1 syndrome, it is felt that the MEN1 gene may act as a tumor suppressor gene and sporadic carcinoid tumors have demonstrated genetic abnormalities in the MEN1 gene [13,14]. 

Though rare, there may also be familial links in the development of carcinoid tumors. Some studies have demonstrated a seemingly familial component to the development of gastrointestinal carcinoid tumors [15-17]. Oliveira et al. in 2001 describe two sets of first-degree relatives who developed pulmonary carcinoid tumors in the absence of clinical features consistent with MEN1 syndrome in what appears to be the first description of familial pulmonary carcinoid tumors [5]. Larger population-based studies have also reported a potential familial link in pulmonary carcinoids, though the relative incidence is lower. While this pattern could reflect true biological mechanisms such as vertical transmission across generations rather than clustering within the same generation, it is more likely attributable to the limited sample size and rarity of pulmonary carcinoid tumors, which reduces the probability of detecting multiple affected relatives of the same degree. Larger datasets or pooled registry analyses will be required to clarify whether this distribution is a reproducible feature of familial aggregation or a sampling artifact. The Swedish Family-Cancer Database includes all persons born in Sweden after 1932, encompassing over 10.2 million people. In 2001, Hemminki and Li reported on the potential familial nature of carcinoid tumors using the database. Among 4,713 parents and 1,933 offspring with carcinoid tumors, there were 17 concordant pairs of parental and offspring carcinoid tumors, with one pair both presenting with pulmonary carcinoid tumors. Though they do not report the standardized incidence ratios (SIRs) for pulmonary carcinoids, there was an overall SIR of 4.31 for offspring of parents who presented with any carcinoid tumor [18]. The lower rate of pulmonary carcinoids among this population may reflect the overall lower incidence of lung cancer in Sweden compared with the United States (17.4/100,000 vs. 35.1/100,000 age-standardized incidence rate, respectively) [19].

In our retrospective population-based description of the incidence of pulmonary carcinoid tumors in the state of Utah, we describe a pattern of incidence that also suggests a potential familial clustering of these rare tumors outside of the MEN1 syndrome. The strengths and limitations of this study should be noted. Limitations include the very small sample sizes, which we can expect to grow only slowly in this resource over time. The UPDB individuals with genealogy data analyzed here represent the descendants of the largely Northern European founders of Utah [20]. This population is representative of much of the United States, but the results reported here should not be extrapolated to other populations. Limitations also include the censoring of cancers diagnosed but not represented in the UPDB because they occurred in another state or before 1966 in Utah. Genealogy data may also be censored and does not always represent biological relationships. The GIF test demonstrated significant excess relatedness overall, with the strongest contributions observed in parent-offspring and avuncular pairs. This distribution may reflect true biological patterns, such as the vertical transmission of heritable risk across generations, or shared exposures within families that persist across multiple generations (e.g., environmental or lifestyle factors). The absence of excess in sibling, cousin, or grandparent-grandchild pairs is more difficult to interpret and may simply reflect the limited number of pulmonary carcinoid cases in our cohort. Larger pooled datasets will be necessary to determine whether this pattern is reproducible or a function of sample size. The study also has many strengths. Cancer data comes from the NCI SEER registry, so cases are histologically confirmed. There are no selection or referral bias in the identification of cases and no ascertainment bias in the identification of relatives. The Utah population has been recognized to be very similar to the US population, and cancer genetic findings originating from studies of Utah pedigrees are well-recognized to be representative of many other populations worldwide.

## Conclusions

Our comparative analysis of the SEER 18 registry demonstrated that, while the incidence of lung cancer overall is significantly lower in the state of Utah compared with the rest of the United States, the incidence of pulmonary carcinoid tumors was similar. We also found significantly increased RRs of developing pulmonary carcinoid in first- and second-degree relatives of 232 probands (RR=6.00 and RR=13.40, respectively). Further genetic analysis would need to be undertaken to exclude the possibility of MEN1 syndrome in these patients; however, it appears strongly suggestive of a potential familial link to pulmonary carcinoid tumors. Whether this is due to a true genetic link, a potential environmental exposure, as all patients are living in the same state, or another unknown link, further investigations are needed. 
